# College students' metacognitive awareness of generative-AI reliance: an experimental study of decision confidence and attribution bias

**DOI:** 10.3389/fpsyg.2026.1926110

**Published:** 2026-09-15

**Authors:** Shaowei Ren

**Affiliations:** School of Civil Engineering and Architecture, Anhui University of Science and Technology, Huainan, Anhui, China

**Keywords:** AI reliance, attribution bias, college students, decision confidence, educational psychology, generative AI, metacognition, trust calibration

## Abstract

**Introduction:**

Generative AI is increasingly used by college students for explanation, evaluation, and decision support, raising the question of whether perceived reliance accurately tracks the extent to which AI advice shapes final judgments.

**Methods:**

In a three-condition between-subjects experiment, 342 undergraduate students completed reasoning and information-evaluation tasks under independent decision-making, open multi-turn ChatGPT support, or the same ChatGPT support with a brief metacognitive reflection prompt. Participants reported initial and final answers and confidence, and AI-assisted chat logs were coded for the final recommendation, its correctness, and behavioral reliance.

**Results:**

Open ChatGPT support increased final confidence and was associated with 62.4% acceptance of incorrect AI advice. Reflection reduced incorrect-advice acceptance to 39.7% (OR = 0.40, 95% CI [0.28, 0.56], *p* < 0.001), improved awareness calibration (0.59 vs. 0.41, *p* < 0.001), and reduced the attribution-bias index (0.21 vs. 0.42, *p* = 0.002). Recommendation accuracy was comparable between the two AI-assisted groups.

**Discussion:**

Brief metacognitive reflection improved immediate discrimination between useful and misleading AI advice without producing blanket rejection of AI. The exploratory association between calibration and overreliance is not interpreted as causal mediation, and the findings concern bounded academic decision tasks rather than long-term learning.

## 1 Introduction

Generative artificial intelligence (AI) has rapidly entered higher education as a tool for writing, explanation, brainstorming, coding, translation, and decision support. For college students, systems based on large language models are no longer peripheral technologies; they increasingly function as readily available cognitive partners that can produce fluent answers, justify recommendations, and present expert-like reasoning. This development creates opportunities for personalized learning and cognitive scaffolding, but it also raises a psychological question that is still insufficiently examined: do students remain aware of the extent to which generative AI shapes their own thinking?

Existing debates on generative AI in education often focus on academic integrity, writing quality, assessment design, and institutional policy. These issues are important, but they do not fully capture the metacognitive consequences of AI-assisted decision-making. Metacognition refers to people's knowledge and regulation of their own cognition, including their ability to monitor uncertainty, evaluate evidence, and adjust strategies when knowledge is incomplete (Flavell, 1979; Nelson and Narens, 1990; Koriat, 1997). When students use generative AI for academic or everyday decisions, they may not simply receive information. They may also recalibrate confidence, shift responsibility, and reinterpret success or failure in relation to the AI system. In this sense, generative AI can influence both first-order decisions and second-order judgments about knowledge, agency, and responsibility.

The present study uses the term metacognitive dependence as an integrative conceptual label for a specific mismatch: students' observable reliance on generative-AI advice may exceed, or otherwise diverge from, their awareness of how strongly that advice shaped their judgment. The term is not treated here as a validated unitary latent trait. Rather, it organizes a set of theoretically related but distinct indicators—behavioral reliance, perceived reliance, decision confidence, and attributional responsibility—that together describe how students monitor AI-assisted decision making. This distinction separates the present focus from simple frequency of AI use. A student may use AI often while accurately recognizing its influence and critically evaluating its advice; conversely, brief use may be consequential when AI-generated reasoning is adopted without corresponding awareness or scrutiny.

Decision confidence and attribution bias provide complementary indicators of this monitoring problem, but they are not assumed to be interchangeable manifestations of a single psychometric construct. Decision confidence reflects how certain individuals feel about the correctness or adequacy of their judgments. Confidence is adaptive when it tracks accuracy, but potentially misleading when it rises without a corresponding improvement in performance. Generative AI may increase confidence because its outputs are fluent, structured, and apparently authoritative even when the content is incomplete or incorrect. Attribution bias concerns how people explain success and failure. In AI-assisted tasks, students may claim internal credit for correct answers while assigning responsibility for errors to AI advice or task difficulty. Examining these indicators alongside direct measures of perceived and behavioral reliance allows the study to distinguish what students do with AI advice from what they believe AI contributed to their decisions.

Research on automation bias and trust in automation has long shown that humans may overuse automated recommendations, especially when systems appear reliable or when users face uncertainty (Parasuraman and Riley, 1997; Lee and See, 2004; Parasuraman and Manzey, 2010). Research on algorithm appreciation and algorithm aversion further suggests that people may either defer to algorithms or reject them depending on prior experience, perceived expertise, and error visibility (Dietvorst et al., 2015; Logg et al., 2019). However, generative AI differs from traditional automation in ways that may intensify metacognitive effects. It produces natural language explanations, adapts to prompts, and often presents answers with a conversational tone. These features can make AI advice feel less like a mechanical output and more like a knowledgeable collaborator.

For college students, this creates a distinctive educational concern. Higher education aims not only to produce correct answers, but also to cultivate independent reasoning, intellectual humility, self-regulation, and responsibility for learning. If generative AI increases confidence without improving monitoring accuracy, students may become less able to distinguish between understanding and assisted performance. If students attribute success to themselves and failure to AI, they may also fail to learn from mistakes. Therefore, the psychological consequences of AI support should be evaluated not only through performance outcomes, but also through metacognitive awareness and attributional patterns.

The present study addresses three gaps in the literature. First, prior work on AI in education has often examined attitudes, usage intention, academic integrity, or learning efficiency, with less attention to whether students' self-reported reliance corresponds to their observed use of AI advice. Second, studies of automation bias usually focus on compliance or accuracy, whereas fewer studies jointly examine behavioral reliance, metacognitive monitoring, confidence, and responsibility attribution during generative-AI interaction. Third, intervention evidence remains limited regarding whether a brief pre-decision reflection prompt can reduce acceptance of misleading AI advice without inducing blanket rejection of useful AI support.

To address these gaps, we designed an experimental study in which college students completed reasoning and information-evaluation tasks under three conditions: independent decision-making, open multi-turn ChatGPT support, and open multi-turn ChatGPT support accompanied by metacognitive reflection. The study examined whether generative-AI support changes decision confidence, whether perceived reliance corresponds to behavioral reliance, whether reflection reduces acceptance of incorrect AI advice, and whether attributional patterns differ across conditions. Random assignment permits causal interpretation of condition effects on directly observed outcomes; analyses relating awareness calibration to incorrect-advice acceptance are treated separately because those variables are not temporally or operationally independent.

Based on metacognitive monitoring theory, automation-bias research, and attribution theory, we tested four condition-effect hypotheses. H1 predicted that open ChatGPT support would increase final decision confidence relative to independent decision-making. H2 predicted substantial behavioral reliance under open ChatGPT support, with the reflection condition showing lower acceptance of incorrect AI advice. H3 predicted that metacognitive reflection would improve correspondence between perceived and behavioral reliance. H4 predicted that open ChatGPT support would produce stronger self-serving attribution bias than the same ChatGPT support accompanied by metacognitive reflection; the independent condition served as a descriptive source-level benchmark where applicable. In addition, an exploratory indirect-effect analysis examined whether variation in awareness calibration statistically covaried with the reflection-related difference in incorrect-advice acceptance. This exploratory analysis was not used to establish a causal mediation mechanism.

## 2 Related work

### 2.1 Generative AI in higher education

Generative AI has quickly become a major issue in higher education because it can produce text, code, explanations, summaries, and feedback at a speed and fluency that exceed most earlier educational technologies. Scholars have emphasized both the pedagogical potential and the risks of these systems. On the positive side, generative AI may support individualized tutoring, rapid feedback, language learning, and access to explanations for students with diverse learning needs (Kasneci et al., 2023; Tlili et al., 2023; Shahzad et al., 2025). On the negative side, it may encourage superficial engagement, weaken independent reasoning, increase academic misconduct, and blur the boundary between student work and machine-generated output (Zhai et al., 2024; Larson, 2024; Lee et al., 2025).

Recent empirical studies show that students' attitudes toward generative AI are generally favorable but mixed. Students often report perceived benefits such as availability, time saving, personalized feedback, and support for writing or brainstorming, while also expressing concerns about factual accuracy, academic integrity, overreliance, privacy, and the devaluation of independent learning (Chan and Hu, 2023; Blahopoulou and Ortiz-Bonnin, 2025; Kim et al., 2025; Maxwell et al., 2025). Cross-national and discipline-sensitive studies further indicate that acceptance of ChatGPT and generative-AI tools varies according to perceived usefulness, habit, self-efficacy, institutional support, and disciplinary expectations (Strzelecki, 2024; Faraon et al., 2025; Alghamdi, 2025).

Recent work has begun to examine these risks in terms of cognitive dependence rather than use frequency alone. A systematic review of AI dialogue systems concluded that overreliance may affect students' decision-making, critical thinking, and analytical reasoning when students accept AI-generated recommendations without adequate verification (Zhai et al., 2024). Survey and experimental-adjacent evidence further suggests that confidence in generative AI may reduce perceived critical-thinking effort, whereas students' own self-confidence and verification strategies are associated with more active evaluation of AI output (Lee et al., 2025). Studies of university students also indicate a paradoxical pattern in which generative-AI use can increase perceived efficiency or self-efficacy while simultaneously strengthening technological dependence or false self-efficacy (Zhang and Xu, 2025; Jia et al., 2025; Kruger and Dunning, 1999).

Many educational discussions focus on whether students should be allowed to use AI and how institutions should detect or regulate such use. These questions are necessary, but they may understate the psychological changes that occur during AI-assisted learning. A student may submit original work while still relying heavily on AI explanations to decide what is true, what is important, or how confident they should feel. Recent work therefore argues that the educational value of generative AI depends less on access alone than on whether students use it to construct and critique knowledge rather than outsource reasoning, and on whether students remain sufficiently risk-aware to avoid academically dishonest or unreflective use (Pallant et al., 2025; Costa and Murphy, 2025; Ortiz-Bonnin and Blahopoulou, 2025). Thus, a psychological analysis of generative AI should include not only observable use behavior, but also the student's monitoring of uncertainty, confidence, and responsibility.

### 2.2 Metacognition and cognitive offloading

Metacognition includes both knowledge about cognition and regulation of cognition (Flavell, 1979; Dunlosky and Metcalfe, 2009; Schraw and Dennison, 1994). In learning contexts, metacognitive monitoring helps students judge whether they understand material, whether they need additional information, and whether a strategy is working. Accurate monitoring is crucial for self-regulated learning because it guides study allocation and error correction. When monitoring is inaccurate, students may become overconfident, stop learning too early, or misjudge the quality of their own understanding.

Generative AI may alter metacognitive monitoring by encouraging cognitive offloading. Cognitive offloading refers to the use of external tools to reduce internal cognitive demands (Risko and Gilbert, 2016). External support is not inherently harmful; notebooks, calculators, search engines, and peer discussion can all support cognition. However, offloading becomes educationally problematic when students lose track of what is internally understood and what is externally supplied. Prior work on the Internet's effects on perceived knowledge suggests that access to external information can inflate people's estimates of their own knowledge (Sparrow et al., 2011; Fisher et al., 2015). Generative AI may intensify this effect because it provides synthesized explanations rather than isolated information.

### 2.3 Automation bias, trust, and AI reliance

Automation bias occurs when people give excessive weight to automated suggestions, sometimes overlooking contradictory evidence or their own correct judgment (Parasuraman and Riley, 1997; Parasuraman and Manzey, 2010). Trust in automation is adaptive when it is calibrated to system competence, but maladaptive when users overtrust unreliable systems or underuse reliable systems (Lee and See, 2004). In AI-assisted decision-making, the challenge is not to maximize trust, but to support appropriate reliance.

Generative AI complicates appropriate reliance because its outputs are not only recommendations, but also explanations. A fluent explanation may increase perceived credibility even when the underlying reasoning is weak. In educational tasks, this creates the possibility that students accept AI advice not because they have evaluated it, but because the response appears coherent. The present study therefore distinguishes AI reliance as behavior, overreliance as acceptance of incorrect AI advice, trust calibration as evaluation of system competence, and metacognitive awareness as the accuracy with which students recognize AI's influence on their own decisions.

### 2.4 Conceptual boundaries: reliance, calibration, and metacognitive awareness

The framework distinguishes several neighboring concepts that are often conflated in discussions of AI use. Automation bias refers to excessive weighting of automated recommendations, especially when users accept erroneous advice (Parasuraman and Riley, 1997; Parasuraman and Manzey, 2010). Overreliance is therefore treated in the present study as a behavioral outcome, operationalized most directly as acceptance of an incorrect final ChatGPT recommendation. Cognitive offloading refers more broadly to transferring cognitive work to an external resource (Risko and Gilbert, 2016); offloading can be efficient and does not by itself imply poor monitoring. Trust calibration concerns whether confidence in a system is appropriate to the system's competence (Lee and See, 2004), whereas metacognitive monitoring accuracy concerns whether people accurately assess their own cognitive state and behavior (Nelson and Narens, 1990; Koriat, 1997).

The present study focuses on the last distinction: whether students accurately recognize the influence that AI advice has on their own final decisions. Accordingly, “metacognitive dependence” is used as a descriptive theoretical framework for a perceived–behavioral reliance mismatch rather than as evidence of a new unitary trait. Behavioral reliance, awareness calibration, confidence, and attribution are analyzed separately. Evidence that these measures change together is informative about the broader framework, but it is not taken as psychometric proof that they form a single latent construct.

### 2.5 Attribution bias in AI-assisted decisions

Attribution theory explains how individuals interpret the causes of success and failure (Weiner, 1985). A robust finding in social psychology is that people often display self-serving bias, attributing success to internal factors such as ability or effort and attributing failure to external factors such as task difficulty or situational constraints (Miller and Ross, 1975). In AI-assisted tasks, attribution becomes more complex because students can allocate responsibility among themselves, the AI system, task difficulty, and external circumstances.

Attribution matters for learning because it influences future motivation and strategy use. If students credit themselves for AI-assisted success, they may overestimate their independent understanding. If they blame AI for failure without examining their own monitoring process, they may fail to learn from errors. Therefore, attribution bias provides a complementary window into responsibility monitoring: it shows how students allocate causal credit and blame after accepting, rejecting, or using AI advice.

## 3 Methodology

### 3.1 Design overview

The study used a between-subjects experimental design with three conditions: independent decision-making, open multi-turn ChatGPT support, and open multi-turn ChatGPT support with metacognitive reflection. Participants completed a set of reasoning and information-evaluation tasks. Each task required an initial answer and confidence rating before any AI interaction. Participants in the two AI conditions then engaged in a real-time ChatGPT consultation and could ask follow-up questions before submitting their final answer. Participants in the reflection condition additionally completed a brief metacognitive prompt after the ChatGPT interaction and before providing their final answer. After final responses, all participants received feedback and completed attribution ratings.

The design allowed the study to test whether open generative-AI interaction changed confidence and reliance, and whether reflection improved awareness of AI reliance under a more naturalistic AI-use setting. The independent condition provided a baseline for confidence and attribution without AI support. The open ChatGPT condition captured AI-assisted decision-making through student-initiated multi-turn ChatGPT interaction. The reflection condition tested whether a minimal intervention could improve metacognitive monitoring during realistic AI use.

### 3.2 Participants

A total of 360 undergraduate students were recruited from Anhui University of Science and Technology through course announcements and online campus notices. Data were collected during the spring semester between March and April 2026 through Wenjuanxing (Questionnaire Star), a university-approved online survey-experiment platform. Recruitment notices described the project as a study of decision-making and digital learning support. Participants received a small non-performance-based course-participation credit or equivalent research-participation acknowledgment; the incentive was unrelated to task accuracy, AI agreement, or completion speed.

A priori sample-size planning used a one-way analysis of variance with three groups as an omnibus benchmark for between-condition effects. A minimum sample of approximately 337 participants was estimated to provide 80% power to detect a small-to-medium condition effect (*f* = 0.17) at α = 0.05, and the recruitment target was increased to 360 to allow for exclusions. The a priori planning analysis did not designate a single primary endpoint; the study was organized around four theoretically specified condition-effect hypotheses. Acceptance of incorrect AI advice is treated as the focal behavioral outcome because it most directly indexes maladaptive reliance on erroneous AI recommendations. Because this outcome was analyzed with a logistic mixed-effects model, the ANOVA calculation should not be interpreted as model-specific power for that outcome or for the exploratory indirect-effect analysis. A design-based sensitivity analysis was therefore conducted for the focal logistic mixed model (see Analytic Strategy).

Exclusion rules were specified in the analysis log before hypothesis testing. Participants were excluded if they failed either of two embedded attention-check items, omitted more than 20% of task or scale responses, or completed the study in less than one-third of the median completion time. Eighteen participants met at least one exclusion criterion. The final analytic sample included 342 participants, with 114 participants in each experimental condition. Participants were between 18 and 24 years old (*M* = 20.21, *SD* = 1.34); 207 identified as female, 132 as male, and 3 selected another gender category or preferred not to report. The sample included students from humanities and social sciences (39.5%), science and engineering (35.1%), business and management (15.5%), and other fields (9.9%).

Inclusion criteria were current undergraduate enrollment, age of at least 18 years, and regular access to digital learning tools. Participants were informed that the study concerned decision-making and digital support in learning contexts rather than an evaluation of personal ability. Participation was voluntary, and all participants provided written informed consent before beginning the experiment. The protocol was reviewed and approved by the Ethics Committee of Anhui University of Science and Technology (approval no. AUST-00286). The study involved minimal risk because tasks were educational, responses were anonymized before analysis, and no sensitive personal information was required.

### 3.3 Procedure

Participants completed the study online in a controlled survey-experiment environment. After informed consent, participants reported demographic information, prior frequency of generative-AI use, self-rated AI literacy, academic self-efficacy, and task familiarity. They were then randomly assigned to one of the three experimental conditions by the survey platform using a block-randomization procedure that kept group sizes approximately equal during data collection. The median completion time was approximately 43 min. Participants were instructed to complete the task individually without using external search engines or additional AI tools other than the study-provided ChatGPT interface.

For each task, participants first selected an initial answer and rated their confidence on a 0–100 scale. Participants in the independent condition then proceeded directly to a final answer and final confidence rating. Participants in the two AI conditions opened a browser-based ChatGPT interface provided through the experimental platform after the initial answer. They copied the task stem and answer options into ChatGPT using a short starter prompt and could ask up to two follow-up questions, allowing a maximum of three interaction turns for each task. The final AI recommendation used for analysis was the last explicit answer choice or recommendation provided by ChatGPT before the participant submitted the final answer. In the reflection condition, participants also responded to three metacognitive prompts after the ChatGPT interaction and before finalizing their answers: (a) “What is my own reason for the answer before relying on AI?”; (b) “Which part of the AI explanation is supported by evidence rather than fluency?”; and (c) “What would make me reject the ChatGPT recommendation?” After the final answer, participants received correctness feedback and rated the causes of the outcome.

Two attention-check items were embedded in the procedure. The first was an instruction-compliance item that asked participants to select a specified response option. The second was a procedure-recall item asking whether ChatGPT interaction had been available during the preceding task block. These checks were used only for data-quality screening and were not included in the substantive analyses.

### 3.4 Experimental conditions

Table 1 summarizes the experimental conditions.

**Table 1:** Experimental conditions and core procedures.

Condition	Decision support	Core metacognitive requirement
Independent decision	Participants completed tasks without AI advice.	Initial and final confidence ratings were collected.
Open ChatGPT support	Participants interacted with ChatGPT after the initial answer and could ask up to two follow-up questions.	Participants reported final answer and confidence after the open AI consultation.
ChatGPT support with reflection	Participants completed the same open ChatGPT consultation.	Participants evaluated their own understanding, AI trustworthiness, and reasons for accepting or rejecting the ChatGPT recommendation before the final answer.

The AI interaction was useful but imperfect. Because participants engaged in open multi-turn ChatGPT consultations, the exact wording and sequence of AI responses varied across participants. To evaluate whether the two AI-assisted conditions received comparable advice quality, each final ChatGPT recommendation was coded against the task answer key and its accuracy was summarized by condition and task category (Table 2). Across AI-assisted task-level observations, the final ChatGPT recommendation was correct in 66.7% of codable trials and incorrect in 33.3%. Incorrect recommendations typically reflected plausible reasoning errors, such as overgeneralizing from a graph, overlooking a base-rate cue, or treating a weak source as more credible than warranted. Crucially, recommendation accuracy was comparable across the open ChatGPT and reflection conditions, reducing the possibility that condition differences in incorrect-advice acceptance were produced simply by unequal exposure to erroneous recommendations.

**Table 2:** Accuracy of final ChatGPT recommendations by condition and task category.

Condition	Analytical reasoning	Data interpretation	Information credibility	Overall
Open ChatGPT support	596/912 (65.4%)	610/912 (66.9%)	608/912 (66.7%)	1,814/2,736 (66.3%)
ChatGPT support with reflection	604/912 (66.2%)	616/912 (67.5%)	614/912 (67.3%)	1,834/2,736 (67.0%)
Combined	1,200/1,824 (65.8%)	1,226/1,824 (67.2%)	1,222/1,824 (67.0%)	3,648/5,472 (66.7%)

Entries are correct final ChatGPT recommendations divided by codable recommendations, with percentages in parentheses. A logistic mixed-effects exposure check with participant and task random intercepts showed no condition difference in recommendation accuracy (β = 0.03, *SE* = 0.06, *z* = 0.57, *p* = 0.57); condition-by-task-category interactions were also not statistically significant (all Holm-adjusted *p*>0.50).

### 3.5 Task materials and open ChatGPT interaction

The task set included three categories: analytical reasoning, data interpretation, and information credibility evaluation. Analytical reasoning tasks required participants to solve short logic or probability problems. Data interpretation tasks required reading small tables or graphs and selecting the best conclusion. Information credibility tasks required evaluating the reliability of brief claims that resembled academic or public-information contexts. Each task had one objectively correct answer and three distractors.

Task materials were reviewed for clarity and difficulty before data collection by two trained research assistants familiar with educational psychology and digital learning research. The 24 tasks were arranged in a randomized order for each participant. To reduce item-specific confounding, task category and expected difficulty were balanced across the experiment. Participants in the two AI-assisted conditions used a browser-based ChatGPT interface provided through the experimental platform. The model label recorded in the experiment log was GPT-5.4 (OpenAI), and data collection using this configuration occurred from 9 March–18 April 2026. Each task was initiated in a fresh task-specific conversation so that prior task content did not carry forward. The standardized starter prompt was the only study-provided task instruction; participants could then ask up to two follow-up questions. In the study configuration, web browsing, persistent memory, file upload, code execution, and other external tool functions were disabled, and no study-specific system instruction or custom GPT configuration was applied beyond the standardized starter prompt. The experimental interface did not expose sampling parameters such as temperature or top-*p* to participants.

Unlike a fixed-advice design, the model was queried dynamically during participant sessions. This choice increased ecological validity but also makes exact response replication impossible because hosted generative-AI systems can change over time. For reproducibility, the Supplementary Materials therefore report the model label and access dates, the system and tool configuration, the complete starter prompt and reflection prompts, the task set and answer keys, the session protocol, anonymized examples of coded interactions, and the coding rule used to identify the final AI recommendation.

To make the open interaction comparable across participants while preserving ecological validity, each AI-assisted task began with the same starter prompt: “Please help me solve this task. I will provide the question and four answer options. Choose the single best answer (A–D), explain your reasoning briefly, and mention any uncertainty if the answer is not clear.” Participants could then ask up to two additional follow-up questions, such as asking for clarification, requesting an alternative explanation, or challenging the ChatGPT response. They were not required to accept the ChatGPT recommendation, and they were reminded that their final answer should reflect their own judgment.

All AI-interaction transcripts were stored with anonymous participant and task identifiers. Two trained coders independently extracted the final explicit ChatGPT recommendation for each AI-assisted trial and coded whether it matched the objective answer key. Inter-coder agreement was high (Cohen's κ = 0.91); disagreements were resolved by discussion using the coding rule that the last explicit answer choice before the participant's final response counted as the final AI recommendation. Fewer than 1% of AI-assisted transcripts initially required coder adjudication because the final recommendation was not immediately explicit; after adjudication using the pre-specified last-explicit-recommendation rule, codable final recommendations were retained for the recommendation-accuracy analyses reported here. The complete task materials, starter prompts, reflection prompts, attention-check items, scale items, chat-log coding rules, variable-coding rules, exclusion criteria, and statistical model specifications are provided in the Supplementary materials.

### 3.6 Measures

#### 3.6.1 Decision confidence

Decision confidence was measured before and after decision support. Initial confidence was the participant's confidence in the first answer. Final confidence was the participant's confidence in the final answer. Confidence change was computed as final confidence minus initial confidence. Confidence accuracy was assessed by comparing confidence ratings with actual correctness.

#### 3.6.2 Behavioral reliance on AI

Behavioral reliance was measured in the AI conditions from the coded ChatGPT transcripts. At the trial level, AI-consistent responding was coded 1 when the participant's final answer matched the final coded ChatGPT recommendation and 0 otherwise. Participant-level behavioral reliance was the proportion of codable AI-assisted trials on which the final answer matched the ChatGPT recommendation,


$$\begin{array}{l} BR_{i}=\frac{1}{n_{i}}\overset{n_{i}}{\underset{j=1}{\sum }}I\left(Final_{ij}=AI_{ij}\right). \end{array}$$ (1)

Behavioral reliance was calculated as shown in Equation 1. Answer revision toward AI advice was coded separately as 1 when a participant's initial answer differed from the final ChatGPT recommendation but the final answer matched it. Acceptance of incorrect AI advice was coded as 1 when the final answer matched a final ChatGPT recommendation that was objectively incorrect. The latter is the focal behavioral indicator of maladaptive reliance because it requires observable adoption of inaccurate advice rather than reliance in general.

#### 3.6.3 Perceived reliance on AI

Perceived reliance was measured after each AI-assisted task using four study-specific items designed to capture participants' immediate perception of AI influence: “The AI advice influenced my final answer,” “I relied on the AI explanation when making my final judgment,” “My confidence changed because of the ChatGPT response,” and “I would probably have answered differently without the AI advice.” Items were rated on a 1–7 scale and averaged so that higher scores indicated stronger perceived reliance. Internal consistency was high (Cronbach's α = 0.88). These items were used as an episode-specific self-report indicator and were not treated as a validated trait measure of dependence.

#### 3.6.4 Awareness calibration

Awareness calibration was defined as correspondence between perceived reliance and behavioral reliance rather than as a separate latent trait. For participant *i*, perceived reliance (*PR*_*i*_), and behavioral reliance (*BR*_*i*_) were standardized across participants in the two AI-assisted conditions, and absolute discrepancy was computed as


$$\begin{array}{l} D_{i}=|z\left(PR_{i}\right)-z\left(BR_{i}\right)|. \end{array}$$ (2)

$$\begin{array}{l} AC_{i}=1-\frac{D_{i}-D_{\min}}{D_{\max}-D_{\min}}, \end{array}$$ (3)

Absolute discrepancy and rescaled awareness calibration were computed using Equations 2 and 3. So that higher values indicated closer perceived–behavioral correspondence.

Because absolute discrepancy treats underestimation and overestimation as equivalent, a directional signed discrepancy was also analyzed:


$$\begin{array}{l} SC_{i}=z\left(PR_{i}\right)-z\left(BR_{i}\right), \end{array}$$ (4)

Signed discrepancy was computed using Equation 4. Where negative values indicate underestimation of behavioral reliance and positive values indicate overestimation. Finally, correspondence was examined without constructing a discrepancy index by estimating a condition-by-behavior interaction predicting perceived reliance at the task level. This slope-based analysis tests whether self-reported reliance more closely tracks AI-consistent responding under reflection. Because self-reports can diverge from underlying cognitive processes (Nisbett and Wilson, 1977), these analyses are interpreted as complementary indicators of monitoring correspondence rather than proof of a unitary metacognitive-dependence construct.

#### 3.6.5 Attribution bias

After receiving outcome feedback, participants rated the extent to which the outcome was due to their own ability, their effort, AI advice, and task difficulty on a 1–7 scale. The same four attribution items were administered in all three conditions to preserve an identical response format. In the independent condition, the AI-advice item served only as a zero-exposure validity check because no AI recommendation had been available. The primary H4 attribution-bias comparison therefore used the two AI-assisted conditions, in which all four attribution sources were substantively applicable. Internal attribution was computed as *INT* = (*Ability*+*Effort*)/2, and external attribution as *EXT* = (*AI Advice*+*Task Difficulty*)/2. For each participant in the AI-assisted conditions, the self-serving attribution-bias index was operationalized as the mean of internal-over-external credit after correct responses and external-over-internal blame after incorrect responses:


$$\begin{array}{l} AB_{i}=\frac{1}{2}\left[\left(INT_{i,correct}-EXT_{i,correct}\right)+\left(EXT_{i,incorrect}-INT_{i,incorrect}\right)\right]. \end{array}$$ (5)

The self-serving attribution-bias index was calculated using Equation 5. Higher values therefore indicate a stronger pattern of internal credit for success combined with external attribution for failure. The independent condition was retained as a descriptive benchmark for the attribution sources that were genuinely available in all conditions (ability, effort, and task difficulty), but its structurally inapplicable AI-advice item was not used to support the primary H4 inference.

Because a composite index can depend on a particular scoring choice, two complementary sensitivity analyses were conducted. First, the four raw attribution ratings were inspected in a source-level mixed-effects analysis, with the independent-condition AI-advice rating interpreted only as a zero-exposure check. Second, within the two AI-assisted conditions, AI-advice attribution was analyzed separately from task-difficulty attribution after incorrect outcomes. These decomposed analyses test whether the H4 condition contrast is visible in the underlying ratings rather than being created by the composite formula.

#### 3.6.6 Control variables

Control variables included prior generative-AI use frequency, self-rated AI literacy, academic self-efficacy, task familiarity, gender, year of study, and discipline group. AI literacy was measured using six items assessing perceived ability to evaluate AI outputs, detect unsupported claims, and understand basic limitations of generative AI (α = 0.86). Academic self-efficacy was measured using five items adapted from self-efficacy research (Bandura, 1997) (α = 0.83). Task familiarity was measured with three items asking whether participants had previously encountered similar reasoning, data interpretation, or credibility-evaluation tasks (α = 0.79).

### 3.7 Analytic strategy

The hypothesis-directed condition-effect analyses addressed H1–H4 using models matched to the level at which each outcome was defined. Trial-level outcomes were analyzed with mixed-effects models containing participant and task random intercepts, whereas participant-level composite outcomes were analyzed at the participant level without duplicating the same composite score across tasks. The focal behavioral outcome was acceptance of an incorrect final ChatGPT recommendation among participants in the two AI-assisted conditions. No causal mechanism was assigned to the awareness-calibration variable because perceived reliance was measured after the decision episode and the calibration score incorporates behavioral reliance.

Before testing incorrect-advice acceptance, we evaluated whether the two AI-assisted conditions were exposed to comparable ChatGPT recommendation quality. Recommendation correctness was modeled with a logistic mixed-effects model including condition, task category, their interaction, and participant and task random intercepts. This exposure analysis directly tests whether any reflection effect could be attributed to different rates or task distributions of incorrect AI advice.

For binary outcomes such as acceptance of incorrect AI advice, logistic mixed-effects models were used. The logistic mixed-effects specification is given in Equation 6.


$$\begin{array}{l} \text{logit}\left(P\left(Y_{ij}=1\right)\right)=\beta_{0}+\beta_{1}Reflection_{i}+\beta_{2}{\text{X}}_{i}+u_{i}+v_{j}, \end{array}$$ (6)

where *Y*_*ij*_ denotes whether participant *i* accepted an incorrect ChatGPT recommendation on task *j*, *Reflection*_*i*_ distinguishes the reflection from the open-ChatGPT condition, **X**_*i*_ represents covariates, and *u*_*i*_ and *v*_*j*_ are participant and task random intercepts. Odds ratios and 95% confidence intervals are reported for focal comparisons.

The participant-level awareness-calibration score was analyzed with a participant-level linear model in the two AI-assisted conditions. It was supplemented by two analyses designed to avoid loss of directional information. Signed discrepancy distinguished underestimation from overestimation of reliance. In addition, a task-level correspondence model tested whether the association between AI-consistent responding and perceived reliance differed by condition. The task-level correspondence model is given in Equation 7.


$$\begin{array}{l} z\left(PR_{ij}\right)=\beta_{0}+\beta_{1}AIConsistent_{ij}+\beta_{2}Reflection_{i}\\ \;+\beta_{3}\left(AIConsistent_{ij}\times Reflection_{i}\right)+u_{i}+v_{j}+\epsilon_{ij}. \end{array}$$ (7)

A positive interaction indicates stronger perceived–behavioral correspondence under reflection.

For attribution, the participant-level composite index was used for the primary H4 comparison between the two AI-assisted conditions; this comparison was conducted at the participant level rather than by repeating composite scores across tasks. Source-level analyses were used as scoring-sensitivity checks, and the independent-condition AI-advice item was treated only as a zero-exposure validity check rather than as substantively equivalent AI attribution. Continuous predictors were standardized before model estimation. Multiple-comparison p-values were adjusted using the Holm procedure where multiple pairwise contrasts were evaluated; the reported 95% confidence intervals are unadjusted intervals for the corresponding focal estimates. Statistical significance was evaluated at *p* < 0.05, and effect sizes are reported for focal tests.

The a priori ANOVA-based sample-size calculation was an omnibus planning benchmark rather than a power calculation for the mixed logistic model. We therefore conducted a design-based simulation sensitivity analysis using the observed group sizes (114 participants in each AI-assisted condition), approximately 1,824 incorrect-advice trial observations, participant- and task-level clustering, and two-sided α = 0.05. Across 1,000 simulated datasets, the design reached approximately 80% power for a reflection effect of about OR ≤ 0.62; the observed focal effect (OR = 0.40) was larger than this sensitivity threshold.

Finally, an exploratory indirect-effect analysis was conducted for the two AI-assisted conditions. Participant-clustered bootstrap confidence intervals with 5,000 resamples were used to quantify whether reflection-related differences in awareness calibration statistically covaried with incorrect-advice acceptance. Because the mediator contains a behavioral-reliance component and was assessed after the relevant decision behavior, the indirect effect is interpreted as associational rather than as evidence that awareness calibration causally mediated the reflection effect. The a priori planning analysis was not designed for this exploratory indirect effect, and no confirmatory power claim is made for it.

## 4 Results

### 4.1 Data quality and manipulation checks

Completion time, attention-check performance, and item-level missingness were inspected before hypothesis testing. The exclusion criteria removed 18 participants, resulting in a balanced final sample across conditions. Missing item responses were rare (less than 1% of task-level observations) and were handled by listwise deletion for the corresponding task-level model. Randomization checks indicated no significant condition differences in age, gender distribution, discipline group, prior generative-AI use, AI literacy, academic self-efficacy, or task familiarity (all *p*>0.10).

Manipulation checks supported both the AI-support manipulation and the reflection manipulation. Perceived exposure to external advice differed strongly across conditions, $F_{\left(2,339\right)}=790.15,p<0.001,\eta_{p}^{2}=0.82$. Participants in the open ChatGPT condition (*M* = 6.05, *SD* = 0.76) and the ChatGPT-with-reflection condition (*M* = 6.01, *SD* = 0.81) reported substantially higher exposure than participants in the independent condition (*M* = 2.06, *SD* = 1.02); the two AI-assisted conditions did not differ significantly from each other (*p* = 0.70). The chat logs also confirmed that participants engaged with the open AI interface: the mean number of ChatGPT turns per task was 2.34 (*SD* = 0.51) in the open ChatGPT condition and 2.41 (*SD* = 0.48) in the reflection condition. In addition, the reflection manipulation increased engagement time on AI-assisted trials. Participants in the reflection condition spent longer per AI-assisted trial (*M* = 104.7 seconds, *SD* = 35.8) than participants in the open ChatGPT condition (*M* = 86.4 seconds, *SD* = 31.2), *t*_(226)_ = 4.11, *p* < 0.001, *d* = 0.55, 95% CI for the mean difference [9.5, 27.1]. These checks indicate that participants noticed and used the ChatGPT interaction and that the reflection prompt produced the intended additional metacognitive processing.

### 4.2 Comparability of ChatGPT recommendation accuracy

Table 2 shows the distribution of correct and incorrect final ChatGPT recommendations across the two AI-assisted conditions and the three task categories. Recommendation accuracy was 66.3% in the open ChatGPT condition and 67.0% in the reflection condition. A logistic mixed-effects model with participant and task random intercepts found no evidence of a condition difference in recommendation accuracy (β = 0.03, *SE* = 0.06, *z* = 0.57, *p* = 0.57). Condition-by-task-category interactions were also nonsignificant after Holm adjustment (all adjusted *p*>0.50). Thus, the reflection and open-ChatGPT groups were exposed to closely comparable rates and task distributions of correct and incorrect final AI recommendations.

### 4.3 Descriptive statistics

Table 3 presents descriptive statistics for the main study variables. Open ChatGPT support increased final confidence relative to independent decision-making. However, the open ChatGPT condition also showed the highest rate of accepting incorrect AI advice. The reflection condition showed lower overreliance and stronger awareness calibration than the open ChatGPT condition.

**Table 3:** Descriptive statistics by experimental condition.

Variable	Independent decision	Open ChatGPT support	ChatGPT support with reflection
Final accuracy (%)	65.8 (14.7)	72.4 (13.5)	75.1 (12.7)
Initial confidence (0–100)	66.9 (13.2)	67.2 (12.9)	66.7 (13.1)
Final confidence (0–100)	68.4 (13.6)	76.1 (12.1)	72.3 (12.6)
Confidence change	1.5 (7.9)	8.9 (8.4)	5.6 (8.1)
AI recommendation accuracy (%)	–	66.3	67.0
Acceptance of incorrect AI advice (%)	–	62.4 (24.1)	39.7 (21.9)
Behavioral reliance	–	0.59 (0.22)	0.47 (0.21)
Perceived reliance	–	5.23 (1.18)	4.89 (1.12)
Awareness calibration	–	0.41 (0.17)	0.59 (0.15)
AI-specific attribution bias index	–	0.42 (0.54)	0.21 (0.48)

Values are means with standard deviations in parentheses unless otherwise indicated. Awareness calibration was scaled from 0 to 1, with higher values indicating better correspondence between perceived and behavioral reliance. The AI-specific attribution-bias index is reported only for the two AI-assisted conditions because the independent condition had no AI recommendation; source-level attribution ratings for the independent condition are retained as a descriptive benchmark.

### 4.4 Correlation analysis

Table 4 reports the correlation matrix for the AI-assisted conditions. Behavioral reliance was positively associated with final confidence and overreliance. Awareness calibration was negatively associated with acceptance of incorrect AI advice and attribution bias. These associations indicate that closer perceived–behavioral correspondence co-occurred with lower incorrect-advice acceptance and lower attribution bias; because the variables were measured within the same decision episode, the correlations are not interpreted causally.

**Table 4:** Correlation matrix for AI-assisted participants.

Variable	1	2	3	4	5
1. Final confidence	1.00				
2. Behavioral reliance	0.36^***^	1.00			
3. Incorrect AI acceptance	0.29^***^	0.48^***^	1.00		
4. Awareness calibration	–0.12	–0.31^***^	–0.44^***^	1.00	
5. Attribution bias	0.22^**^	0.27^***^	0.34^***^	–0.38^***^	1.00

Correlations are based on participants in the two AI-assisted conditions. ^**^p < 0.01, ^***^ *p* < 0.001.

### 4.5 Condition effects on decision confidence

The first hypothesis predicted that open ChatGPT support would increase final decision confidence. The condition effect on final confidence was significant, $F_{\left(2,339\right)}=10.34,p<0.001,\eta_{p}^{2}=0.06$. Holm-adjusted pairwise contrasts showed that students in the open ChatGPT condition reported higher final confidence than students in the independent condition (*M*_*diff*_ = 7.7, 95% CI [4.37, 11.03], adjusted *p* < 0.001). The reflection condition also showed higher final confidence than the independent condition (*M*_*diff*_ = 3.9, 95% CI [0.57, 7.23], adjusted *p* = 0.044), but lower confidence than the open ChatGPT condition (*M*_*diff*_ = −3.8, 95% CI [–7.13, –0.47], adjusted *p* = 0.044).

This pattern supports H1. Generative-AI support increased confidence, but metacognitive reflection attenuated the magnitude of this confidence increase. The reflection condition did not eliminate the confidence increase; instead, it produced a more restrained shift in subjective certainty.

Figure 1 complements the summary statistics by showing the full distribution of confidence before and after decision support. The initial-confidence distributions were closely aligned across the three groups, indicating that the later confidence divergence was unlikely to be driven by baseline differences. After open ChatGPT interaction, the open ChatGPT condition shifted upward and became concentrated at higher confidence levels, whereas the reflection condition showed a more moderate shift. This distributional pattern indicates that reflection did not simply suppress confidence; rather, it constrained the confidence boost produced by AI advice and kept students' final judgments closer to their initial metacognitive baseline.

**Figure 1:**
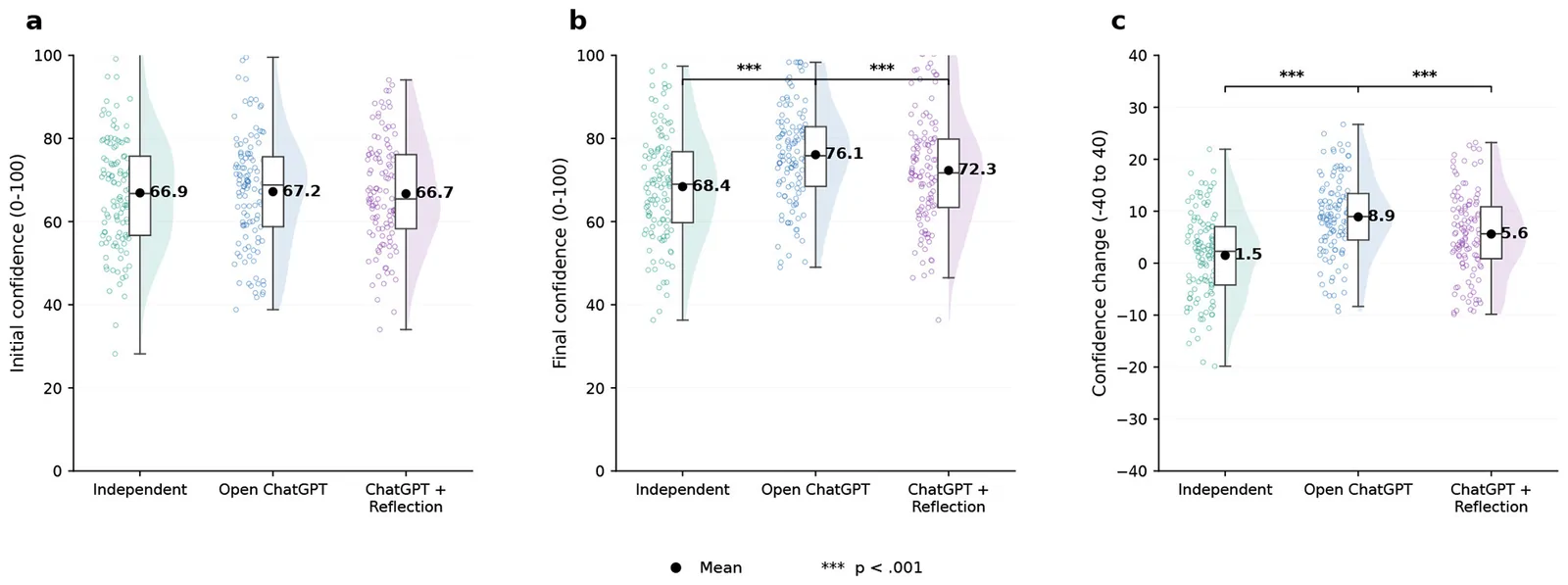
Distributional changes in decision confidence across experimental conditions. **(a)** Initial confidence was comparable across the independent, open ChatGPT, and ChatGPT-with-reflection conditions. **(b)** Final confidence increased most strongly under open ChatGPT support, while the reflection condition showed an intermediate pattern. **(c)** Confidence change was largest in the open ChatGPT condition and was attenuated by metacognitive reflection. Points show participant-level observations, boxplots summarize median and interquartile range, violin densities show distribution shape, and black dots indicate group means.

### 4.6 Condition effects on AI overreliance

H2 predicted that open ChatGPT support would increase behavioral reliance, especially acceptance of incorrect AI advice. Among participants in the AI-assisted conditions, open ChatGPT support produced a higher rate of accepting incorrect ChatGPT recommendations than ChatGPT support with reflection (62.4% vs. 39.7%). A logistic mixed-effects model confirmed this difference (β = −0.92, *SE* = 0.18, *z* = −5.11, *p* < 0.001, odds ratio = 0.40, 95% CI [0.28, 0.56]), indicating that reflection substantially reduced the odds of accepting incorrect AI advice. This condition effect was estimated after the exposure check showed comparable AI-recommendation accuracy across the two AI-assisted groups and remained substantively unchanged when task category and recommendation-exposure terms were included as covariates.

Answer revision patterns showed a similar result. Students in the open ChatGPT condition revised their answers toward AI advice on 58.6% of trials, whereas students in the reflection condition did so on 46.8% of trials. The difference was most pronounced for incorrect AI advice, suggesting that reflection did not merely reduce all AI use; it specifically helped students resist misleading advice.

Figure 2 further clarifies that the reflection effect was selective rather than a general rejection of AI. When the final ChatGPT recommendation was correct, both AI-assisted groups frequently aligned their final answers with the recommendation across analytical reasoning, data interpretation, and information-credibility tasks. The larger separation appeared when the final ChatGPT recommendation was incorrect: the open ChatGPT group continued to follow misleading recommendations, whereas the reflection group showed a consistent downward shift across all task domains. This pattern strengthens the interpretation that reflection improved discriminative reliance–students remained able to use helpful AI advice while becoming less vulnerable to plausible but incorrect ChatGPT recommendations.

**Figure 2:**
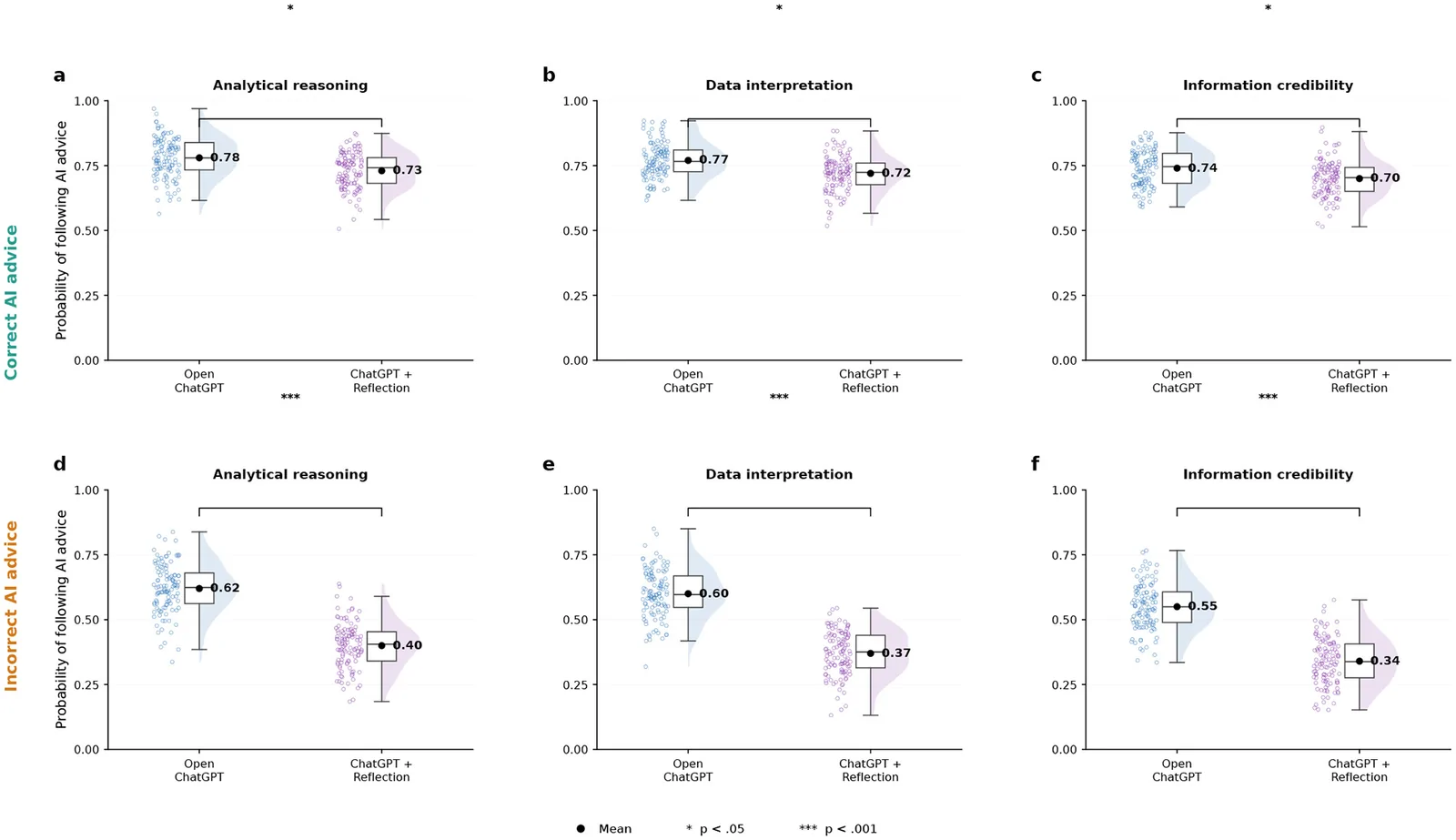
AI-consistent final responses by advice correctness and task category. Panels **(a-c)** show trials in which the coded final ChatGPT recommendation was correct, and panels **(d-f)** show trials in which the coded final ChatGPT recommendation was incorrect. Columns correspond to analytical reasoning, data interpretation, and information-credibility tasks. The open ChatGPT condition and ChatGPT-with-reflection condition showed similar high alignment with correct advice, but reflection markedly reduced alignment with incorrect advice. Points show participant-level tendencies within task categories, boxplots summarize dispersion, violin densities show distribution shape, and black dots indicate means.

### 4.7 Awareness calibration

H3 predicted that metacognitive reflection would improve correspondence between perceived and behavioral reliance. The reflection condition showed higher absolute calibration than the open ChatGPT condition (*M* = 0.59, *SD* = 0.15 vs. *M* = 0.41, *SD* = 0.17), *t*_(226)_ = 8.48, *p* < 0.001, *d* = 1.12, 95% CI for the mean difference [0.14, 0.22]. Trial-level associations were also stronger in the reflection condition (*r* = 0.51) than in the open ChatGPT condition (*r* = 0.24).

The directional signed-discrepancy analysis showed why the absolute score differed between conditions. The open ChatGPT condition had a negative mean signed discrepancy (*M* = −0.22, *SD* = 0.71), indicating that behavioral reliance tended to exceed perceived reliance, whereas the reflection condition was close to zero (*M* = −0.02, *SD* = 0.66); the between-condition difference was statistically significant, *t*_(224.8)_ = 2.20, *p* = 0.029, *d* = 0.29.

A slope-based mixed-effects analysis provided a second check that did not rely on the absolute-discrepancy index. AI-consistent responding was positively associated with perceived reliance in both conditions, but this correspondence was stronger under reflection. The condition-by-AI-consistent-response interaction was positive (β = 0.27, *SE* = 0.07, *p* < 0.001); standardized simple slopes were approximately β = 0.24 in the open ChatGPT condition and β = 0.51 in the reflection condition.

Together, these analyses support a narrower interpretation than a unitary “dependence” construct: reflection improved the observable correspondence between what students reported about AI influence and how often their final answers aligned with AI advice. The signed analysis further suggests that the weaker calibration under open ChatGPT support was characterized primarily by underestimation rather than by symmetric over- and underestimation.

Figure 3 visualizes this correspondence at the participant level. In the open ChatGPT condition, perceived reliance and behavioral reliance were weakly aligned and observations were more dispersed from the diagonal reference. In the reflection condition, the distribution was more concentrated around the diagonal. The figure therefore illustrates a perceived–behavioral correspondence difference; it is not interpreted as evidence that calibration temporally mediated later decision behavior.

**Figure 3:**
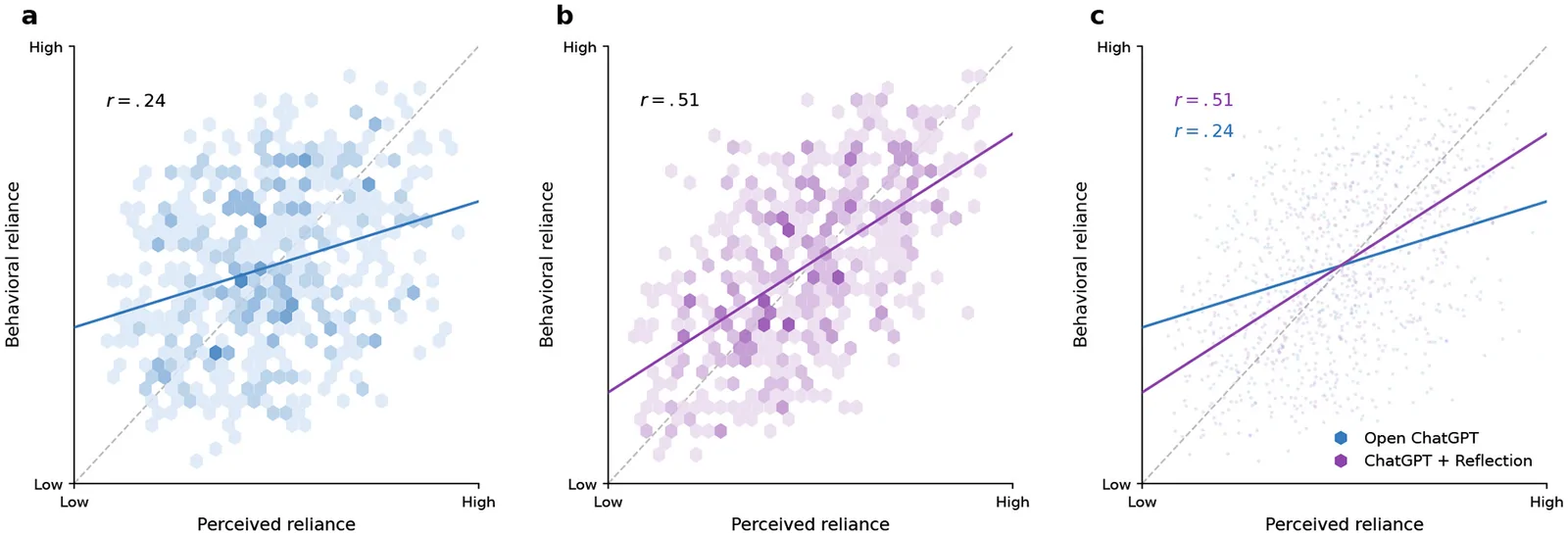
Awareness calibration between perceived reliance and behavioral reliance. **(a)** In the open ChatGPT condition, perceived and behavioral reliance showed a weaker association and wider dispersion from the diagonal calibration line. **(b)** In the ChatGPT-with-reflection condition, perceived reliance tracked behavioral reliance more closely. **(c)** The combined view shows a stronger perceived–behavioral correspondence pattern and denser alignment near the diagonal in the reflection condition. The dashed diagonal represents perfect correspondence between perceived and behavioral reliance, and fitted lines summarize condition-specific calibration trends.

### 4.8 Attribution bias

H4 predicted that open ChatGPT support would produce stronger self-serving attribution bias than ChatGPT support with reflection. Because the AI-advice source was substantively applicable only when AI advice had actually been available, the primary composite-index test was restricted to the two AI-assisted conditions. The open ChatGPT condition showed a higher attribution-bias index (*M* = 0.42, *SD* = 0.54) than the reflection condition (*M* = 0.21, *SD* = 0.48), *t*_(222.94)_ = 3.10, *p* = 0.002, *d* = 0.41, 95% CI for the mean difference [0.08, 0.34]. This result supports H4: reflection reduced the AI-specific self-serving attribution pattern relative to open ChatGPT support.

The independent condition was retained as a descriptive benchmark for attribution sources that were meaningful without AI support. In particular, its AI-advice item was treated only as a zero-exposure validity check and was not used to claim that an AI-specific composite had the same psychological meaning in the independent and AI-assisted conditions. Figure 4 therefore emphasizes the source-level pattern rather than treating the independent-condition AI-advice cell as substantively equivalent to AI attribution in the two AI-assisted groups.

**Figure 4:**
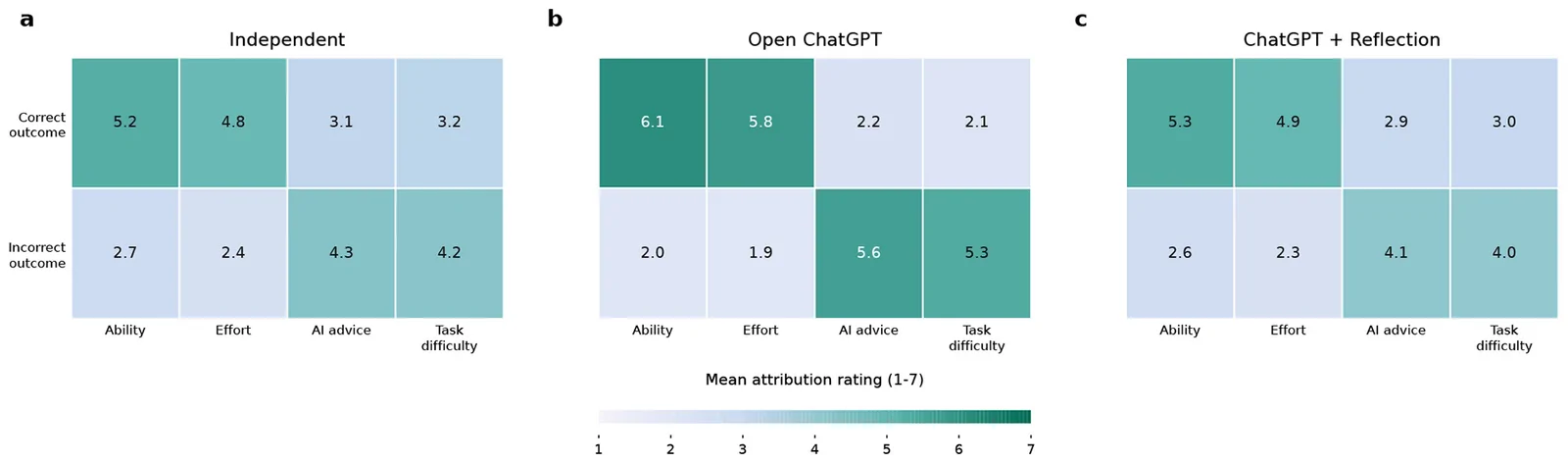
Source-level attribution patterns after correct and incorrect outcomes. Panels **(a-c)** show the Independent, Open ChatGPT, and ChatGPT + Reflection conditions, respectively. Heatmaps show mean ratings for ability, effort, AI advice, and task difficulty on a 1–7 scale. In the independent condition, the AI-advice cell is a zero-exposure validity check because no AI recommendation was available and is not interpreted as substantively equivalent to AI attribution in the assisted conditions. The open ChatGPT condition showed the strongest internal-credit/external-blame asymmetry, whereas the reflection condition showed a more balanced source-level profile.

The scoring-sensitivity analyses reached the same substantive conclusion. A mixed-effects model of the four raw attribution sources showed a significant condition-by-outcome-correctness-by-attribution-source interaction [likelihood-ratio χ^2^(6) = 22.1, *p* = 0.001]. In this model, the independent-condition AI-advice rating functioned only as the expected zero-exposure reference. More importantly for H4, within the incorrect-outcome subset of the two AI-assisted conditions, attribution specifically to AI advice was higher in the open ChatGPT condition than in the reflection condition (estimated mean difference = 0.46 points on the 1–7 scale, 95% CI [0.16, 0.76], Holm-adjusted *p* = 0.004), whereas the condition contrast for task-difficulty attribution was smaller. Thus, the H4 conclusion did not depend on collapsing AI advice and task difficulty into a single external-attribution score.

### 4.9 Covariate-adjusted models

Table 5 presents covariate-adjusted models for the three focal outcomes that map cleanly onto a single regression specification. Model 1 predicts task-level final confidence, Model 2 predicts trial-level acceptance of incorrect AI advice among AI-assisted participants, and Model 3 predicts the participant-level awareness-calibration score. The attribution-bias hypothesis is reported separately above because its primary composite contrast is restricted to the two AI-assisted conditions and is supported by source-level sensitivity analyses. All models include controls for prior AI use, AI literacy, academic self-efficacy, task familiarity, gender, year of study, and discipline group.

**Table 5:** Covariate-adjusted models for focal confidence, overreliance, and calibration outcomes.

Predictor	Final confidence	Incorrect AI acceptance	Awareness calibration
Open ChatGPT support	0.38^***^ (0.07)	–	–
ChatGPT support with reflection	0.19^*^ (0.07)	–0.92^***^ (0.18)	0.47^***^ (0.08)
Prior AI use	0.11^*^ (0.05)	0.18^*^ (0.08)	–0.09 (0.06)
AI literacy	0.07 (0.05)	–0.21^**^ (0.08)	0.16^*^ (0.06)
Academic self-efficacy	0.14^*^ (0.06)	–0.07 (0.07)	0.12 (0.06)
Task familiarity	0.17^**^ (0.06)	–0.12 (0.08)	0.09 (0.06)
Controls included	Yes	Yes	Yes
Model level	Trial	Trial	Participant
Random intercepts	Participant/task	Participant/task	None
Participants	342	228	228
Observations	8,208	1,824	228
Model fit	$R_{m}^{2}=0.18$	AUC = 0.74	–

Standardized coefficients are reported for continuous outcomes. Model 2 reports log-odds from a logistic mixed-effects model predicting acceptance of incorrect AI advice. Models 1 and 2 use participant and task random intercepts because their outcomes are defined at the task/trial level. Model 3 analyzes one awareness-calibration score per AI-assisted participant and therefore uses a participant-level linear model rather than repeating the same composite score across tasks. Continuous predictors were standardized before estimation. Standard errors are shown in parentheses. ^*^ *p* < 0.05, ^**^ *p* < 0.01, ^***^ *p* < 0.001.

The covariate-adjusted results were consistent with the primary condition-effect analyses. Random assignment supports a causal interpretation of the reflection-condition coefficient for directly observed experimental outcomes such as incorrect-advice acceptance. Open ChatGPT support increased final confidence relative to the independent condition, whereas reflection reduced incorrect-advice acceptance and was associated with higher participant-level awareness calibration. AI literacy was associated with lower incorrect-advice acceptance, although this observational covariate association is not interpreted causally. The attribution findings are interpreted from the hypothesis-directed AI-assisted contrast and the source-level analyses reported in the preceding subsection; the participant-level attribution composite is not treated as a task-level repeated outcome.

### 4.10 Exploratory indirect-effect analysis

Table 6 reports the exploratory bootstrap indirect-effect results for the two AI-assisted conditions. The exploratory indirect-effect analysis focused on the two AI-assisted conditions. Reflection was associated with higher awareness calibration (*a* = 0.47, *p* < 0.001). Awareness calibration was negatively associated with acceptance of incorrect AI advice (*b* = −0.17, *p* < 0.001) after adjustment for condition and covariates. The product-of-coefficients indirect association was *ab* = −0.081, with a participant-clustered bootstrap 95% CI [–0.129, –0.039]. The adjusted direct reflection coefficient remained statistically different from zero (*c*′ = −0.61, *p* < 0.01). The indirect association is not labeled “partial mediation” because the calibration score partly incorporates behavioral reliance and was assessed after the decision episode.

**Table 6:** Exploratory bootstrap indirect-effect results.

Path	Estimate	SE	95% CI lower	95% CI upper
Reflection → awareness calibration	0.47	0.08	0.31	0.63
Awareness calibration → incorrect AI acceptance	–0.17	0.04	–0.25	–0.10
Indirect effect	–0.081	0.023	–0.129	–0.039
Direct effect	–0.61	0.19	–0.98	–0.24
Total effect	–0.69	0.18	–1.05	–0.34

The exploratory model was estimated for the two AI-assisted conditions (*N* = 228) using 5,000 participant-clustered bootstrap resamples. Effects involving incorrect AI acceptance are reported on the log-odds scale. The indirect estimate quantifies statistical covariation and is not interpreted as evidence of temporally ordered causal mediation.

The indirect association is consistent with the possibility that perceived–behavioral correspondence and incorrect-advice acceptance changed together under reflection, but it does not establish that improved calibration caused the reduction in overreliance. A causal mediation claim would require a mediator that is measured before the behavioral outcome and is operationally independent of that outcome. The remaining direct condition effect is compatible with additional processes such as increased scrutiny of AI explanations, but those mechanisms were not directly isolated in the present study.

### 4.11 Robustness checks

Table 7 summarizes the robustness and specification checks for the reflection effect. Several robustness and specification checks were conducted. First, results were substantively unchanged when confidence change rather than final confidence was used as the outcome. Second, the reduction in incorrect AI acceptance remained significant when only high-difficulty tasks were analyzed and when task category was included explicitly. Third, the awareness conclusion was supported both by signed discrepancy and by the condition-specific perceived–behavioral correspondence slope, reducing reliance on a single absolute-discrepancy score. Fourth, the attribution conclusion remained visible when the four attribution sources were modeled directly rather than collapsed into a composite. Fifth, excluding participants with very high prior AI use did not alter the direction or significance of the focal reflection effect. The alternative correspondence analyses do not remove the temporal-overlap limitation of the exploratory indirect-effect model, which is therefore retained only as an associational analysis.

**Table 7:** Robustness checks for the reflection effect.

Specification	Outcome	Reflection effect	Significance
Baseline model	Incorrect AI acceptance	–0.92	*p* < 0.001
High-difficulty tasks only	Incorrect AI acceptance	–0.88	*p* < 0.001
Signed discrepancy	Reliance underestimation	0.20	*p* = 0.029
Correspondence-slope interaction	Perceived reliance	0.27	*p* < 0.001
Source-level attribution model	Attribution source pattern	–	*p* = 0.001
Excluding high-frequency AI users	Incorrect AI acceptance	–0.95	*p* < 0.001
Confidence-change outcome	Confidence change	-0.29	*p* < 0.01

## 5 Discussion

The present study examined college students' metacognitive awareness of generative-AI reliance. Open ChatGPT support increased immediate confidence but was also accompanied by substantial acceptance of incorrect recommendations and stronger self-serving attribution. The randomized reflection condition reduced incorrect-advice acceptance, improved perceived–behavioral reliance correspondence, and attenuated attribution bias. These condition effects are directly supported by the experimental design; by contrast, associations between calibration and incorrect-advice acceptance are interpreted cautiously because the measures overlap operationally and were not temporally ordered as mediator and outcome.

### 5.1 Generative AI as a metacognitive context

The findings support the view that generative AI is not merely an external information tool. It also functions as a metacognitive context that changes how students experience their own knowledge. Students in the open ChatGPT condition became more confident after interacting with ChatGPT, even when some advice was incorrect. The increase in confidence was therefore not reducible to accuracy alone; it also reflected a shift in subjective certainty following AI interaction. The pattern is consistent with the possibility that fluency, coherence, and apparent completeness can serve as confidence cues, although the present design did not independently manipulate linguistic fluency and therefore cannot isolate that explanation.

The reflection condition showed a different pattern. Participants continued to engage with the AI interface, but their confidence increase was smaller and their acceptance of incorrect AI advice was lower. This suggests that reflection did not create blanket distrust of AI. Instead, it supported more discriminating reliance. In educational terms, this is important because the goal is not to discourage all AI use, but to help students use AI while maintaining responsibility for evaluation.

### 5.2 Metacognitive awareness of AI reliance

The study contributes to the literature by distinguishing behavioral reliance from awareness of reliance. The open ChatGPT condition showed weaker correspondence between self-reported and observed AI influence, and the signed-discrepancy analysis indicated that this mismatch was characterized mainly by underestimation. We use the phrase metacognitive dependence to describe this broader monitoring problem, not to claim that the present indicators constitute a single latent psychological trait. This distinction matters because reliance can be appropriate when it is recognized and critically regulated, whereas unrecognized reliance may make it harder for students to decide when verification is needed.

The awareness-calibration results support this distinction at the level of observable correspondence. Reflection increased alignment between perceived reliance and AI-consistent responding and reduced the average tendency to underestimate behavioral reliance. These findings show improved monitoring correspondence during the bounded task episode; they do not establish a stable metacognitive trait or a long-term reduction in dependence.

### 5.3 Attribution bias and responsibility in AI-assisted learning

The attribution findings show that responsibility judgments differed across AI-support conditions after outcomes were known. In the open ChatGPT condition, students showed stronger self-serving attribution bias: they were more likely to claim credit for correct responses while assigning responsibility to AI when errors occurred. This pattern is consistent with self-serving attribution, although the present study did not measure delayed learning and therefore cannot determine whether the attribution pattern impaired subsequent learning.

Metacognitive reflection reduced the AI-specific composite attribution-bias score relative to open ChatGPT support, and the source-level sensitivity analysis showed the same direction without relying on the composite scoring rule. The independent condition served only as a descriptive benchmark for attribution sources that were meaningful without AI exposure. One plausible interpretation is that reflection made the student's role in accepting or rejecting AI advice more salient, but this explanatory process was not independently manipulated. The result therefore supports further investigation of agency-focused AI literacy rather than establishing a specific causal attribution mechanism.

### 5.4 Theoretical contributions

This study makes three theoretical contributions. First, it provides an experimental framework for distinguishing behavioral reliance on generative AI from students' metacognitive awareness of that reliance. The framework does not require the indicators to form a unitary latent construct; instead, it separates reliance behavior, perceived reliance, confidence, and attributional responsibility so that their convergence and divergence can be examined directly. Second, it connects metacognitive monitoring with automation bias, cognitive offloading, and attribution theory while preserving the conceptual boundaries among these traditions. This distinction clarifies that accepting incorrect advice is a behavioral overreliance outcome, whereas perceived–behavioral correspondence concerns monitoring accuracy. Third, the randomized intervention provides evidence that a brief reflection prompt can reduce immediate acceptance of incorrect AI advice while simultaneously increasing perceived–behavioral correspondence. The present data do not establish that the latter change causally mediates the former.

These contributions extend educational psychology by moving beyond outcome-only evaluation of AI tools. AI-supported task outcomes, confidence, reliance, and responsibility judgments need not move together. Evaluating AI-supported education therefore benefits from measuring both behavioral outcomes and metacognitive indicators, while claims about durable learning require longitudinal or transfer outcomes not assessed here.

### 5.5 Practical implications

The findings have several implications for higher education. First, the findings suggest that educators may consider complementing permission-oriented AI policies with structured opportunities for critical and reflective use. Second, assignments that permit AI assistance may benefit from asking students to document initial reasoning, identify uncertainty, and explain how AI advice influenced final decisions. Third, AI-literacy programs may benefit from including metacognitive monitoring alongside technical instruction about prompts and model limitations. Fourth, assessment designs can distinguish AI-supported performance from performance demonstrated without assistance when the learning objective requires independent mastery.

These implications do not require complex institutional systems. The reflection prompt used in the present study is brief and could be embedded in learning management systems, writing assignments, laboratory reports, or digital exams. The goal is not to make students distrust AI, but to help them remain aware of their own role in AI-assisted cognition.

### 5.6 Limitations and future directions

Several limitations bound the interpretation of the findings. First, the sample was drawn from one university. Random assignment supports internal causal inference for the experimental condition effects, but it does not establish that effect sizes will generalize to other institutions, cultures, disciplines, or age groups. Second, the study used relatively short reasoning and information-evaluation tasks. The findings therefore concern immediate decision behavior rather than semester-long learning, durable skill acquisition, or transfer after AI support is removed. Third, although participants engaged in real multi-turn ChatGPT interaction, the interaction was bounded by a standardized starter prompt, a maximum number of turns, and a controlled online environment. Hosted generative-AI systems also evolve over time, which makes reporting the exact model label, access dates, session protocol, and coding rules essential for reproducibility.

Fourth, awareness calibration is not operationally independent of the focal behavioral outcome. The calibration index includes behavioral reliance, whereas incorrect-advice acceptance is itself a reliance-related behavior. In addition, perceived reliance was assessed after the AI-assisted decision episode. Consequently, the exploratory indirect-effect analysis cannot establish a temporally ordered causal mediation mechanism. The signed-discrepancy and slope-based correspondence analyses strengthen the descriptive conclusion that reflection improved perceived–behavioral alignment, but they do not solve this temporal limitation. Future experiments should measure prospective reliance expectations or manipulate metacognitive calibration before the final decision so that mediator and outcome are both temporally and operationally distinct.

Fifth, the attribution-bias index is a constructed composite and is substantively comparable only when AI advice was actually available. We therefore restricted the primary composite-index contrast to the two AI-assisted conditions, treated the independent-condition AI-advice item only as a zero-exposure validity check, and supplemented the composite with source-level analyses. Future work should validate attribution measures across settings and examine whether post-outcome attribution predicts later strategy change. Finally, the study does not provide psychometric validation of “metacognitive dependence” as a single latent construct. The term is used as an integrative framework for a mismatch between AI-assisted behavior and awareness of that behavior. Future research could determine whether stable individual differences exist by using multi-wave measurement, latent-variable modeling, convergent and discriminant validity tests, and behavioral calibration tasks that are independent of the outcome being predicted.

Future research should also examine longer academic activities such as writing, research synthesis, programming, and source evaluation; test reflection interventions across multiple institutions; and include delayed retention and transfer assessments. Such designs would clarify whether the immediate monitoring advantages observed here translate into durable learning and self-regulation.

## 6 Conclusion

This study examined college students' metacognitive awareness of generative-AI reliance through decision confidence, incorrect-advice acceptance, perceived–behavioral reliance correspondence, and attribution. Open ChatGPT support increased immediate confidence but was also accompanied by substantial acceptance of incorrect recommendations and stronger self-serving attribution. A brief randomized reflection prompt reduced incorrect-advice acceptance, improved perceived–behavioral correspondence, and attenuated attribution bias during the experimental tasks.

The findings support a bounded conclusion: reflection can improve immediate monitoring and selective use of AI advice in short academic decision tasks. They do not establish metacognitive dependence as a unitary trait, prove a causal mediation pathway through awareness calibration, or demonstrate long-term learning effects. Responsible AI use in higher education may therefore benefit from interventions that make students' own reasoning and criteria for accepting AI advice explicit, while future multi-site, longitudinal, and transfer studies are needed to determine whether these immediate effects persist beyond the experimental setting.

## Data Availability

The raw data supporting the conclusions of this article will be made available by the authors, without undue reservation.

## References

[B1] Alghamdi A. A. (2025). University students' perceptions of generative AI for critical thinking and creativity: The influence of self-efficacy and disciplinary differences. Innov. Educ. Teach. Int. 2025, 1–16. doi: 10.1080/14703297.2025.2600476

[B2] Bandura A. (1997). Self-Efficacy: The Exercise of Control. New York, NY: Freeman.

[B3] Blahopoulou J. Ortiz-Bonnin S. (2025). Student perceptions of chatgpt: Benefits, costs, and attitudinal differences between users and non-users toward ai integration in higher education. Educ. Inf. Technol. 30, 19741–19764. doi: 10.1007/s10639-025-13575-9

[B4] Chan C. K. Y. Hu W. (2023). Students' voices on generative AI: perceptions, benefits, and challenges in higher education. Int. J. Educ. Technol. High. Educ. 20:43. doi: 10.1186/s41239-023-00411-8

[B5] Costa C. Murphy M. (2025). Generative artificial intelligence in education: (what) are we thinking? *Learn. Media Technol*. doi: 10.1080/17439884.2025.2518258. [Epub ahead of print].

[B6] Dietvorst B. J. Simmons J. P. Massey C. (2015). Algorithm aversion: people erroneously avoid algorithms after seeing them err. J. Exper. Psychol. 144, 114–126. doi: 10.1037/xge0000033 25401381

[B7] Dunlosky J. Metcalfe J. (2009). Metacognition. Thousand Oaks, CA: Sage.

[B8] Faraon M. Rönkkö K. Milrad M. Tsui E. (2025). International perspectives on artificial intelligence in higher education: an explorative study of students' intention to use chatgpt across the nordic countries and the usa. Educ. Inf. Technol. 30, 17835–17880. doi: 10.1007/s10639-025-13492-x

[B9] Fisher M. Goddu M. K. Keil F. C. (2015). Searching for explanations: How the internet inflates estimates of internal knowledge. J. Exper. Psychol. 144, 674–687. doi: 10.1037/xge0000070 25822461

[B10] Flavell J. H. (1979). Metacognition and cognitive monitoring: a new area of cognitive-developmental inquiry. Am. Psychol. 34, 906–911. doi: 10.1037/0003-066X.34.10.906

[B11] Jia W. Pan L. Neary S. (2025). Effect of genai dependency on university students' academic achievement: The mediating role of self-efficacy and moderating role of perceived teacher caring. Behav. Sci. 15:1348. doi: 10.3390/bs15101348 PMC12561201 41153138

[B12] Kasneci E. Sessler K. Küchemann S. Bannert M. Dementieva D. Fischer F. . (2023). Chatgpt for good? On opportunities and challenges of large language models for education. Learn. Individ. Differ. 103:102274. doi: 10.1016/j.lindif.2023.102274

[B13] Kim J. Klopfer M. Grohs J. R. Eldardiry H. Weichert J. Cox L. A. . (2025). Examining faculty and student perceptions of generative AI in university courses. Innov. High. Educ. 50, 1281–1313. doi: 10.1007/s10755-024-09774-w

[B14] Koriat A. (1997). Monitoring one's own knowledge during study: a cue-utilization approach to judgments of learning. J. Exper. Psychol. 126, 349–370. doi: 10.1037/0096-3445.126.4.349

[B15] Kruger J. Dunning D. (1999). Unskilled and unaware of it: how difficulties in recognizing one's own incompetence lead to inflated self-assessments. J. Pers. Soc. Psychol. 77, 1121–1134. doi: 10.1037/0022-3514.77.6.1121 10626367

[B16] Larson B. Z. (2024). Critical thinking in the age of generative AI. Acad. Manag. Learn. Educ. 23, 373–378. doi: 10.5465/amle.2024.0338

[B17] Lee H.-P. Sarkar A. Tankelevitch L. Drosos I. Rintel S. Banks R. Wilson N. (2025). “The impact of generative ai on critical thinking: self-reported reductions in cognitive effort and confidence effects from a survey of knowledge workers,” in Proceedings of the 2025 CHI conference on human factors in computing systems, 1–22. doi: 10.1145/3706598.3713778

[B18] Lee J. D. See K. A. (2004). Trust in automation: designing for appropriate reliance. Hum. Factors 46, 50–80. doi: 10.1518/hfes.46.1.50.30392 15151155

[B19] Logg J. M. Minson J. A. Moore D. A. (2019). Algorithm appreciation: people prefer algorithmic to human judgment. Organ. Behav. Hum. Decis. Process. 151, 90–103. doi: 10.1016/j.obhdp.2018.12.005

[B20] Maxwell D. Oyarzun B. Kim S. Bong J. Y. (2025). Generative ai in higher education: demographic differences in student perceived readiness, benefits, and challenges. TechTrends 69, 1248–1259. doi: 10.1007/s11528-025-01109-6

[B21] Miller D. T. Ross M. (1975). Self-serving biases in the attribution of causality: Fact or fiction? Psychol. Bull. 82, 213–225. doi: 10.1037/h0076486

[B22] Nelson T. O. Narens L. (1990). “Metamemory: a theoretical framework and new findings,” in Psychology of learning and motivation, 125–173. doi: 10.1016/S0079-7421(08)60053-5

[B23] Nisbett R. E. Wilson T. D. (1977). Telling more than we can know: verbal reports on mental processes. Psychol. Rev. 84, 231–259. doi: 10.1037/0033-295X.84.3.231

[B24] Ortiz-Bonnin S. Blahopoulou J. (2025). Chat or cheat? Academic dishonesty, risk perceptions, and chatgpt usage in higher education students. Soc. Psychol. Educ. 28:113. doi: 10.1007/s11218-025-10080-2

[B25] Pallant J. L. Blijlevens J. Campbell A. Jopp R. (2025). Mastering knowledge: the impact of generative AI on student learning outcomes. Stud. High. Educ. 51, 714–735. doi: 10.1080/03075079.2025.2487570

[B26] Parasuraman R. Manzey D. H. (2010). Complacency and bias in human use of automation: an attentional integration. Hum. Factors 52, 381–410. doi: 10.1177/0018720810376055 21077562

[B27] Parasuraman R. Riley V. (1997). Humans and automation: use, misuse, disuse, abuse. Hum. Factors 39, 230–253. doi: 10.1518/001872097778543886

[B28] Risko E. F. Gilbert S. J. (2016). Cognitive offloading. Trends Cogn. Sci. 20, 676–688. doi: 10.1016/j.tics.2016.07.002 27542527

[B29] Schraw G. Dennison R. S. (1994). Assessing metacognitive awareness. Contemp. Educ. Psychol. 19, 460–475. doi: 10.1006/ceps.1994.1033

[B30] Shahzad M. F. Xu S. Zahid H. (2025). Exploring the impact of generative AI-based technologies on learning performance through self-efficacy, fairness &ethics, creativity, and trust in higher education. Educ. Inf. Technol. 30, 3691–3716. doi: 10.1007/s10639-024-12949-9

[B31] Sparrow B. Liu J. Wegner D. M. (2011). Google effects on memory: cognitive consequences of having information at our fingertips. Science 333, 776–778. doi: 10.1126/science.1207745 21764755

[B32] Strzelecki A. (2024). Students' acceptance of chatgpt in higher education: an extended unified theory of acceptance and use of technology. Innov. High. Educ. 49, 223–245. doi: 10.1007/s10755-023-09686-1

[B33] Tlili A. Shehata B. Adarkwah M. A. Bozkurt A. Hickey D. T. Huang R. . (2023). What if the devil is my guardian angel: chatGPT as a case study of using chatbots in education. Smart Learn. Environ. 10:15. doi: 10.1186/s40561-023-00237-x

[B34] Weiner B. (1985). An attributional theory of achievement motivation and emotion. Psychol. Rev. 92, 548–573. doi: 10.1037/0033-295X.92.4.548 3903815

[B35] Zhai C. Wibowo S. Li L. D. (2024). The effects of over-reliance on ai dialogue systems on students' cognitive abilities: a systematic review. Smart Learn. Environ. 11:28. doi: 10.1186/s40561-024-00316-7

[B36] Zhang L. Xu J. (2025). The paradox of self-efficacy and technological dependence: unraveling generative AI's impact on university students' task completion. Internet High. Educ. 65:100978. doi: 10.1016/j.iheduc.2024.100978

